# Tubulin tyrosine nitration regulates microtubule organization in plant cells

**DOI:** 10.3389/fpls.2013.00530

**Published:** 2013-12-26

**Authors:** Yaroslav B. Blume, Yuliya A. Krasylenko, Oleh M. Demchuk, Alla I. Yemets

**Affiliations:** Department of Genomics and Molecular Biotechnology, Institute of Food Biotechnology and Genomics, National Academy of Sciences of UkraineKyiv, Ukraine

**Keywords:** microtubules, α-tubulin, detyrosination/tyrosination cycle, nitric oxide signaling, tyrosine nitration, 3-nitrotyrosine, kinesin-8, *Arabidopsis*

## Abstract

During last years, selective tyrosine nitration of plant proteins gains importance as well-recognized pathway of direct nitric oxide (NO) signal transduction. Plant microtubules are one of the intracellular signaling targets for NO, however, the molecular mechanisms of NO signal transduction with the involvement of cytoskeletal proteins remain to be elucidated. Since biochemical evidence of plant α-tubulin tyrosine nitration has been obtained recently, potential role of this posttranslational modification in regulation of microtubules organization in plant cell is estimated in current paper. It was shown that 3-nitrotyrosine (3-NO_2_-Tyr) induced partially reversible *Arabidopsis* primary root growth inhibition, alterations of root hairs morphology and organization of microtubules in root cells. It was also revealed that 3-NO_2_-Tyr intensively decorates such highly dynamic microtubular arrays as preprophase bands, mitotic spindles and phragmoplasts of *Nicotiana tabacum* Bright Yellow-2 (BY-2) cells under physiological conditions. Moreover, 3D models of the mitotic kinesin-8 complexes with the tail of detyrosinated, tyrosinated and tyrosine nitrated α-tubulin (on C-terminal Tyr 450 residue) from *Arabidopsis* were reconstructed *in silico* to investigate the potential influence of tubulin nitrotyrosination on the molecular dynamics of α-tubulin and kinesin-8 interaction. Generally, presented data suggest that plant α-tubulin tyrosine nitration can be considered as its common posttranslational modification, the direct mechanism of NO signal transduction with the participation of microtubules under physiological conditions and one of the hallmarks of the increased microtubule dynamics.

## Introduction

Nitric oxide (NO) is a key player in redox signaling pathways in plant cell revealing concentration-dependent effects—from the mild regulation of morphogenesis to the triggering of the programmed cell death (PCD) events (Neill et al., 2008; Baudouin, 2011). Dinitrogen trioxide (N_2_O_3_), nitrogen dioxide (NO_2_), and highly reactive molecule of nitrogen monoxide (NO) that exist in cell in three interchangeable forms [nitrosonium cation (NO^+^), nitroxyl anion (NO^−^) and free radical (NO^•^)] along with peroxynitrite (ONOO^−^) and S-nitrosothiols (GSNOs) are named reactive nitrogen species (RNS) (Neill et al., 2008). RNS are able to modify numerous proteins affecting their structure, protein–protein interaction and/or function (“loss”/“gain” and the enhanced protein turnover) (Lindermayr et al., 2005; Abello et al., 2009). Direct RNS-mediated signaling is realized by such protein posttranslational modifications as NO-iron heme binding, S-nitrosylation of reduced cysteine and the C-nitration of tyrosine, tryptophan, cysteine and methionine residues (Besson-Bard et al., 2008; Wilson et al., 2008; Blume et al., 2009).

Tyrosine nitration upsides S-nitrosylation is important pathway in redox signaling (Leon et al., 2008; Monteiro et al., 2008). It is a covalent binding of nitro group (−NO_2_) to one of the two equivalent ortho-positions of the phenolic ring of tyrosine residues yielding a modified amino acid 3-nitrotyrosine (3-NO_2_-Tyr) (Leon et al., 2008). The process of tyrosine nitration is supposed to be low-abundant *in vivo*, since proteins usually have approximately 3–4 mol% of Tyr and not all Tyr residues undergo nitration (Abello et al., 2009; Chaki et al., 2009). Importantly, it occurs under physiological conditions *in vivo* and depends on distinct local environment (pH level in microcompartments, activity of antioxidant enzymes, ROS/RNS balance, etc.) and special protein conformation (Aulak et al., 2004; Abello et al., 2009). Tyrosine nitration is supposed to be not a random chemical process, but a highly specific targeted event in cell signaling regulatory posttranslational modification of proteins *pari passu* with S-nitrosylation due to the putative denitrase activity or non-enzymatic mechanisms (Koeck et al., 2004; Besson-Bard et al., 2008; Abello et al., 2009; Blume et al., 2009; Deeb et al., 2013).

For quite a long time, tyrosine nitration was supposed to be an irreversible posttranslational modification disrupting protein structure/function (“guaranteed kiss of death in proteasome”) and relatively reliable biological marker of the nitrosative stress (Corpas et al., 2008), but novel data for its physiological regulatory role, non-related to the specific adverse conditions, are accumulated. Newly discovered nitroproteomes include 21 proteins in sunflower cotyledons (Chaki et al., 2009), 16 proteins in pea roots (Begara-Morales et al., 2013) and 127 proteins in *Arabidopsis* seedlings (Lozano-Juste et al., 2011; Corpas et al., 2013). The identification of potential *in vivo* nitration sites of some *Arabidopsis* proteins is reported (Lozano-Juste et al., 2011). Protein targets of tyrosine nitration could vary between plant species, tissues, developmental stage, eustress/distress conditions, etc. (Corpas et al., 2013). Thus, a stage-specific distribution of cytosolic tyrosine-nitrated proteins in the hypocotyl segments of sunflower seedlings (*Helianthus annuus* L.) during adventitious roots formation reveals a new level of regulation of plant development through this post-translational mechanism (Yadav et al., 2013). Nitrosoproteome of sour orange plants (*Citrus aurantium* L.) is involved into plants acclimation to salinity stress (Tanou et al., 2012). Furthermore, in mammalian cells tyrosine nitration participates in signal transduction (Greenacre and Ischiropoulos, 2001), neuronal differentiation (Giannopoulou et al., 2002; Cappelletti et al., 2004), and embryonic development (Capeletti et al., 2006).

Among RNS downstream targets *in vitro* and *in vivo* are eukaryotic cytoskeletal proteins (for review see Yemets et al., 2011). As in other cases, most of data that tubulin undergoes tyrosine nitration came from mammalian systems (α-tubulin) (Eiserich et al., 1999; Kalisz et al., 2000; Bisig et al., 2002; Chang et al., 2002; Giannopoulou et al., 2002; Peluffo et al., 2004; Capeletti et al., 2006; Phung et al., 2006) and β-tubulin (Banan et al., 2000) after the mimicking of the nitrosative stress conditions. Recently, the basal levels of α-tubulin and four proteins co-precipitated in complex with it (22, 28, 36, and 54 kDa) tyrosine nitration in the extracts of intact *Nicotiana tabacum* Bright Yellow-2 (BY-2) cells were found by Western-blot analysis with further immunoprecipitation of α-tubulin and 3-NO_2_-Tyr (Yemets et al., 2011). Both α-(α3/α5 and α6 chains) and β-(β 1, β 2/3, and β 4 chains) tubulins as well as actins-2 and 7 were identified among immunopurified Tyr-nitrated proteins from *Arabidopsis* seedlings by a shotgun LC-MS/MS approach *in vivo* (Lozano-Juste et al., 2011).

The mechanism of α-tubulin tyrosine nitration is supposed to be different from the direct nitration of Tyr residues in other cytoskeletal proteins. It is highly selective process, since two specific endogenously nitrated Tyr 161 and Tyr 357 of the mammalian α-tubulin have been identified (Tedeschi et al., 2005). Newly synthesized mammalian and plant α-tubulin contains two Tyr residues its C-terminus (Smertenko et al., 1997; Westermann and Weber, 2003). The enzymatic machinery of the mammalian α-tubulin detyrosination/tyrosination cycle includes tubulin-specific carboxypeptidase (TCP) that cleaves Tyr residues from the labile C-tail with the formation of Glu-tubulin and tubulin-specific tyrosine ligase (TTL) that adds non-modified or modified Tyr residues to the initial site (Westermann and Weber, 2003). This evolutionary conservative posttranslational modification regulates the dynamicity/stability of distinct MTs populations as dynamic tyrosinated and relatively stable detyrosinated populations of microtubules can coexist in cell (Westermann and Weber, 2003). It appears that the majority of plant cell microtubules (MTs) are tyrosinated and that only a small subgroup exists in detyrosinated state (Smertenko et al., 1997). Promiscuous substrate specificity of the mammalian TTL favors the incorporation into α-tubulin the modified Tyr such as chlorotyrosine, mono-, and diodotyrosine, antiproloferative agent azatyrosine, fluorotyrosine and, finally, 3-NO_2_-Tyr (Prota et al., 2013). TTL binds nitrated Tyr to α-tubulin both *in vitro* and *in vivo* (Westermann and Weber, 2003) and TTCP removes it as well as unmodified Tyr (Bisig et al., 2002). One homolog of TTL was found in the *Arabidopsis* (Gardiner and Marc, 2003) and *in silico* model of α-tubulin tubulin–TTL-complex has been recently proposed (Prota et al., 2013). However, the reversibility α-tubulin tyrosine nitration is still an open question, because no enzyme (TTCP or denitrase) have been identified in plant cell. It remains to be elucidated, whether TTL non-specifically binds 3-NO_2_-Tyr to α-tubulin or it binds Tyr to α-tubulin and then ONOO^−^ nitrates it in a specific Tyr residue(s). The signaling role of α-tubulin tyrosine nitration could be also revealed in its interference with α-tubulin tyrosine phosphorylation in a competition for the binding sites (Blume et al., 2008).

Numerous reports provide data supporting the reversibility of this modification as it occurs *in vivo* and participates in such physiological processes as normal development of the chicken embryo chorioallantoic membrane (Giannopoulou et al., 2002), cytoskeleton stabilization in differentiating neuronal-like cells (Capeletti et al., 2006), cell cycle progression of vascular smooth muscles cells (Phung et al., 2006) and could play a role of native mechanism of cytoskeletal proteins turnover (Palumbo et al., 2002). 3-NO_2_-Tyr incorporation into α-tubulin C-tail has no detrimental effects neither on dividing epithelial lung carcinoma A549 cells morphology, viability, proliferation and MTs assembly (Bisig et al., 2002), nor on astrocytes cell morphology, growth and survival (Peluffo et al., 2004). Though, in rice and tobacco cells non-reversible incorporation of free 3-NO_2_-Tyr led to the impairment of MTs organization and functions as well as cell division and cell wall deposition (Jovanović et al., 2010).

In this paper we report for the first time the localization of 3-NO_2_-Tyr on mitotic MTs (preprophase bands, mitotic spindles and phragmoplasts) in BY-2 cells under physiological conditions, 3-NO_2_-Tyr effects on MTs organization in *Arabidopsis* root cells as well as on such cytoskeleton-related processes as root growth and differentiation. Moreover, *in silico* spatial reconstruction of kinesin-8 interaction with tyrosinated, detyrosinated and tyrosine nitrated C-terminus of *Arabidopsis* as one of the putative mechanisms of the regulation of MTs dynamics is provided.

## Materials and methods

### Plant material, growth conditions, and chemical treatment

*Arabidopsis thaliana* (L). Heynh. ecotype Columbia (Col-1) WT and ecotype Landsberg erecta (Ler) expressing *gfp-map4* (***m***icrotubule-***a***ssociated ***p***rotein 4) (Mathur and Chua, 2001) seeds were surface sterilized by the immersion in 0.3% sodium hypochlorite for 15 min, rinsed five times in sterile water and placed on a half-strength MS medium (Murashige and Skoog, 1962) including vitamins (Duchefa, Haarlem, Netherlands), supplemented with 10 g L^−1^ sucrose and solidified with 4 g L^−1^ Gelrite (Duchefa), pH 5.7. Seeds were stratified at 4°C for 1 day and then vertically cultivated in a growth chamber at 23°C with 8/16 h photoperiod.

3-Nitrotyrosine (3-NO_2_-Tyr) (Sigma-Aldrich, USA) was dissolved in distilled water with the addition of HCl and used at 1, 5, 10, 50, 100, and 200 μ M concentrations. Since even the slight shift in pH could affect MT organization (Takahashi et al., 2003), controls were treated with 0.5 μM HCl (at the highest solvent concentration to dissolve 3-NO_2_-Tyr completely) or 200 μM L-tyrosine water solutions. Four-day-old seedlings were submerged into the solutions of the abovementioned chemicals for 2-72 h.

*Nicotiana tabacum* BY-2 suspension culture and BY-2 expressing *gfp-mbd* (***m***icrotubule ***b***inding ***d***omain) (Granger and Cyr, 2001) were maintained according to the modified method of Nagata et al. (1992) in 50 ml of growth medium containing 4.4 g/l of basic MS macro- and microsalts, 200 mg/l KH_2_PO_4_, 30 g/l sucrose, 0.2 mg/l 2,4-dichlorophenoxyacetic acid (2.4-D), 1 mg/l thiamine hydrochloride and 10 mg/l MYO-inositol (pH 5.8) sterilized by autoclaving at 120°C for 15 min prior to use. The cells were subcultured by regular transfer of 2 ml of a 7-day-old culture into 50 ml of fresh medium in a 250 ml Erlenmeyer flask under sterile conditions and incubated on a rotary shaker (Heidolph Incubator 1000) at 130 rpm at 28°C in darkness.

Sodium nitroprusside (SNP) (Sigma-Aldrich, USA), a widely used exogenous NO donor, was dissolved in distilled water as a 10 mM stock solution immediately before the experiments avoiding the direct light exposure and used at 200 μM, 1 and 5 mM concentrations. SNP solution inactivated by a day-long exposure of seedlings under a common fluorescent lamp was used as control (Ötvös et al., 2005). Three days-old BY-2 cells in exponential growth phase were treated with either active or inactivated SNP for 3 h.

### *A. thaliana* primary root length measurements and microtubules visualization in *A. thaliana* and by-2 cells

Time-lapse images of growing *A. thaliana* seedling were captured by a Canon Power Shot G6 digital camera (Canon, China) in the macro mode. The increase in root length was measured with Image J software (version 1.44) (http://rsbweb.nih.gov/ij/index.html). Effects of 3-NO_2_-Tyr on roots growth were determined as the percentage ratio between roots length (in mm) at the beginning of the experiments (0 h) to the roots length (in mm) after chemicals treatment for 2, 6, 24, 48, and 72 h. Root growth rates (D) were calculated using the following equation: (L_ev_–L_0_/L_0_) * 100%, where L_0_—initial root length (no treatment); L_ev_—roots length after the treatment (Yemets et al., 2008). Data represent means ± standard error (*SE*) (*n* = 10 of at least three independent experiments). To estimate the statistical significance between means, the data were analyzed by Student's *t*-test.

The fluorescent signal from GFP fused with one of MT-binding domains (Mathur and Chua, 2001) was visualized with Carl Zeiss CLSM 510 META (Carl Zeiss, Jena, Germany) using 63× Plan-Apochromat oil-immersion objective (1.4 NA), excitation 488/543 nm, emission 510/540 nm) in *A. thaliana* roots and BY-2 cells *in vivo*. The viability of *A. thaliana* roots cells after 200 μM 3-NO_2_-Tyr was evaluated using propidium iodide (PI) staining (Sigma-Aldrich, USA). The selected images represent the same results from 10 replicates per one treatment.

The number of dividing BY-2 (GFP-MBD) cells was estimated using 40× EC Plan-Neofluar objective by counting preprophase bands, mitotic spindles and phragmoplasts. To increase reliability of counting, 4',6-diamidino-2-phenylindole (DAPI) (5 μg/l) (Sigma-Aldrich, USA) was added allowing to observe chromosomes during pro-, meta-, ana-, and telophases. Results were expressed as mean ± *SE* (*n* = 1000 cells in visual field in four independent replicates). *P* < 0.001 value of *t*-test was considered significant for mean differences.

### Indirect immunofluorescent microscopy of α-tubulin and 3-NO_2_-Tyr localization in by-2 cells

The optimized protocol was based on Szechyńska-Hebda et al. (2005). For α-tubulin and 3-NO_2_-Tyr immunolocalization intact BY-2 cells were fixed in 4% paraformaldehyde, 0.1% glutaraldehyde in microtubule-stabilizing buffer (MTSB) (50 mM PIPES-KOH, 5 mM EGTA, 5 mM MgSO4), pH 7.0, for 40 min. After rinsing with MTSB with the addition of 0.1% Triton X-100 for three times during for 10 min, BY-2 samples cells were immobilized on 0.1% poly-L-lysine (Sigma-Aldrich, USA)-covered adhesive slides. Cell walls digestion were achieved by 1% cellulase Onozuka R10 (Sigma-Aldrich, USA) and 0.5% Pectolyase (Sigma-Aldrich, USA) solution in MTSB for 15 min. Cooled methanol (−20°C) treatment of cells for 6 min preceded the rinsing in the phosphate-saline buffer (PBS) with the addition of 0.1% Triton X-100. In order to eliminate unspecific binding of antibodies with MTs the samples were exposed to 2% bovine serum albumin (BSA) in PBS for 30 min. Samples were incubated overnight with mouse anti-α-tubulin TU-01(1:50) recognizing the defined epitope (aa 65–97) on N-terminal structural domain of α-tubulin (kindly provided by Dr. P. Dráber, Institute of Molecular Genetics, Prague, Czech Republic) and rabbit anti-3-NO_2_-Tyr antibodies (1:150) (Sigma-Aldrich, USA) diluted in PBS containing 2% (w/v) BSA under the room temperature (RT). Following four times rinsing in MTSB, BY-2 cells were incubated with the secondary FITC-conjugated anti-mouse (1:32) (Sigma-Aldrich, USA) and TRITC-conjugated anti-rabbit (1:70) (Sigma-Aldrich, USA) antibodies diluted in PBS containing 2% (w/v) BSAs supplemented with 2 μM leupeptin (Sigma-Aldrich, USA) in humid chamber under +37°C for 2.5 h. Negative controls of the reaction specificity (no primary antibodies, test for the cross-linkage between inappropriate primary and secondary antibodies were also performed. To counter-stain nuclei, samples were rinsed three times in PBS for 10 min, then DAPI (5 μg/l) solution was pipetted on each slide for 5 min.

After the final four times rinsing with PBS for 10, the slides were covered by microglass and examined with Carl Zeiss CLSM 510 META (Carl Zeiss, Jena, Germany). Images were acquired at the same gain and exposure time using appropriate optical filter sets with a 63× Plan-Apochromat oil immersion objective. FITC emission was observed under the excitation with using the 488 line of the Ar-laser, TRITC—543 nm of He/Ne laser, DAPI—405 nm of UV laser. Emission signals of rhodamine dyes FITC and TRITC were split using META system with short- and long-wave filters BP 505–530 nm and LP 560 nm, respectively. Image assembly and analysis was performed in a software package of CLSM 510 META and Adobe Photoshop CS.

### Computational simulation of plant tubulins and kinesin

Three-dimensional (3D) model of plant kinesin-8 was constructed on the online server SwissModel Workspace (Arnold et al., 2006) using the *A. thaliana* primary sequence (UniProtKB/TrEMBL code: Q9FZ77) and 3LRE crystal of human kinesin-8 motor domain KIF18A (Peters et al., 2010) as a matrix. The full 3D models of α-and β-tubulins from *A. thaliana* were constructed using the online server I-TASSER on the basis of primary sequences P11139 and P12411, respectively (Blume et al., 2010). Overlay of the constructed molecules of *A. thaliana* kinesin-8, α- and β-tubulins with the appropriated structures from 1IA0 crystal (Kikkawa et al., 2001) was performed in Swiss-PdbViewer 4.1.0 software package. Alteration of C-terminal region position of plant α-tubulin and Tyr450 modifications were simulated in the Discovery Studio 3.5 Visualizer company Accelrys^©^ software package.

The optimization of the molecular geometry of the constructed models, molecular dynamics simulation of protein-protein complex and analysis of the results were performed using the specialized modules from Gromacs 4.5.4 software package (Hess et al., 2008; Pronk et al., 2013). Topology data for the unnatural amino acid used for charmm27 force field was obtained from the database SwissSidechain (Gfeller et al., 2013). In order to reproduce the intracellular environment, protein complexes were immersed in water and its volume was determined by the size of the studied macromolecules. Distant electrostatic interactions were calculated by PME method (Particle Mesh Ewald) (Essmann et al., 1995). As one of the conditions of its use for the calculation of the Coulomb interactions is the neutral system status, the nonzero charges of the obtained systems were neutralized by the automatic substitution of random water molecules with sodium and chlorine ions, which amount corresponded to physiological concentration (0.15 mol/L). Optimization of the molecular geometry was carried out by the potential energy minimization by the steep descent algorithm under the maximum steps number of 1000 and gradient 0.1 in the force field charmm27. The quality of the obtained models was estimated on the MolProbity server (Chen et al., 2010).

The process of the molecular dynamics that lasted 20 ns was carried out using *grompp* and *mdrun* modules. To simulate the solvent volume periodic boundary conditions were applied. The temperature of the system was maintained at 310K using the Berendsen thermostat with the interaction time of 0.1 ps. Constant pressure was maintained by an external barostat. Bond lengths between the hydrogen atoms were fixed at the equilibrium levels using the Lincs algorithm (Hess et al., 1997). Error messages in the molecules structure were absent. Analysis of the molecular dynamics was carried out using *g_dist* and *g_hbond* modules. Visualization of all molecules was performed in the Discovery Studio 3.5 Visualizer company Accelrys^©^ software package.

Molecular dynamics calculation was carried out with the assistance of The Ukrainian National Grid (UNG) (http://grid.nas.gov.ua) and IFBG cluster of virtual organization CSLabGrid.

## Results

### 3-nitrotyrosine affects *A. thaliana* primary root growth, morphology, and microtubules organization in root cells

**Figure 1:**
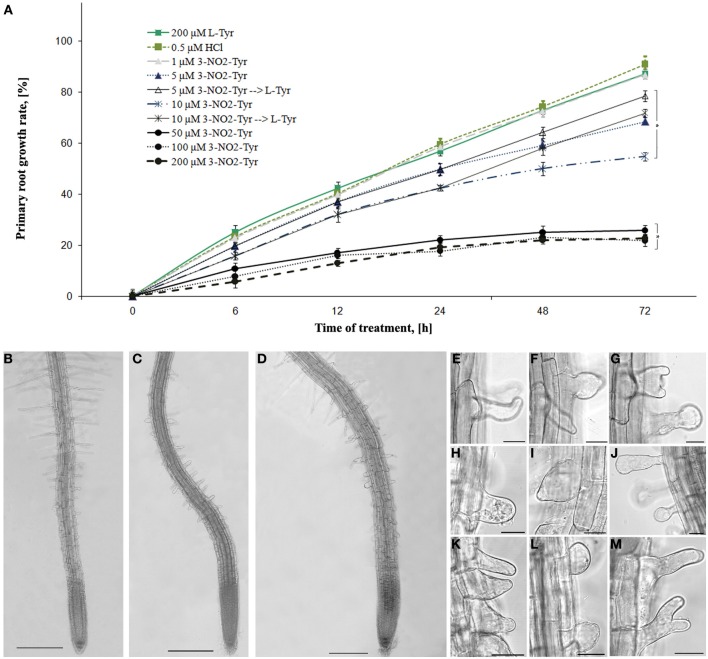
**The effects of 3-NO_2_-Tyr on *A. thaliana* primary roots growth and morphology. (A)** Growth rate of *A. thaliana* roots during 1–200 μM 3-NO_2_-Tyr treatment for 2–72 h. Vertical bars on graphs represent the SE. Columns with asterisk are significantly different (*t*-test, *P* <0.05) from their controls (L-Tyr/HCl); **(B–M)** Morphology of *A. thaliana* primary roots after 24 h of treatment: **(B)** control (200 μM L-Tyr); **(C)** 50 μ M; **(D)** 100 μ M; **(E–M)** Different patterns of the impaired root hairs morphology: **(E–H)** 5 μ M; **(I,J)** 10 μ M; **(K,L)** 50 μM; **(M)** 100 μM. Bar: **(C–E)** = 200 μm; **(F–M)** = 20 μm.

It was found that 3-NO_2_-Tyr (5–200 μM) caused specific *A. thaliana* roots growth inhibition in concentration-dependent manner (Figure 1A).

3-NO_2_-Tyr at concentrations 5–200 μM caused significant (*P* < 0.05) inhibition of root growth reached their maximum value after 24 h of treatment. Thus, the growth rate of 5 and 10 μM 3-NO_2_-Tyr-treated seedlings decreased to 49.7 and 42.3% after 24 h, while of 50, 100, and 200 μM—to 21.9, 17.6, and 19.1%, respectively as compared to L-Tyr/HCl-exposed seedlings (56.8 and 59.4%). Moreover, the part of 5 and 10 μM 3-NO_2_-Tyr-treated seedlings were transferred to 200 μM L-Tyr solution after 24 h and growth of their roots partially recovered at 48 h and 72 h of the experiment, while the growth of seedlings exposed to higher (>10 μM) 3-NO_2_-Tyr concentrations remained stunted (not shown).

The alteration of root differentiation revealed in the formation of numerous ectopic root hairs with the impaired morphology (swelled, branched and/or curved) and stunted growth (Figures 1E–M) as compared to L-Tyr treated seedlings (Figure 1B). Furthermore, slight swelling of epidermal cells of the transition and elongation zones appeared after the seedlings treatment with 100 μM 3-NO_2_-Tyr (Figure 1D), while its lower concentrations (5, 10, and 50 μM) have not affected the polarity of cell growth (Figure 1C).

**Figure 2:**
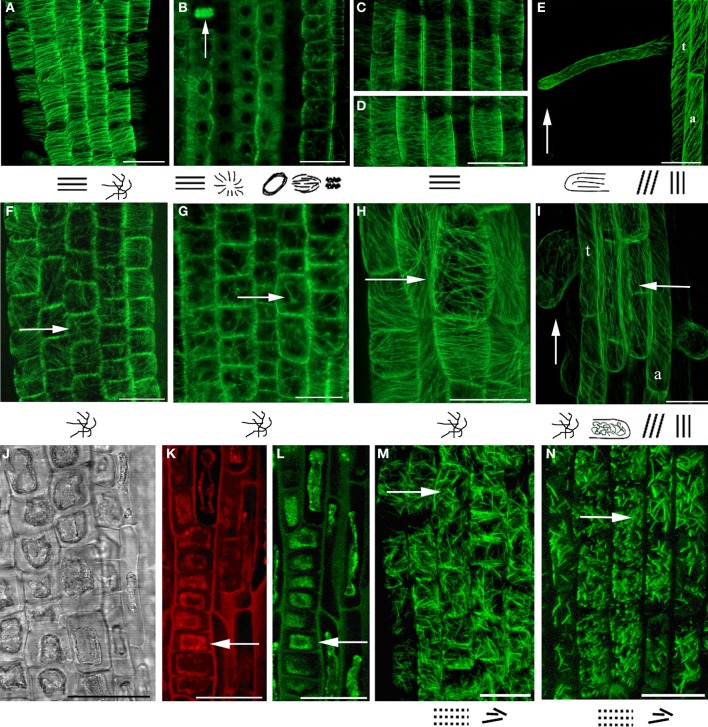
**MTs organization in epidermal cells of *A. thaliana* (GFP–MAP4) primary roots. (A–D)** Control seedlings (L-Tyr, 200 μM): **(A)** root apex; **(B)** meristematic zone; **(C)** transition zone; **(D)** elongation zone; **(E)** trichoblasts (t) and atrichoblasts (a) of the differentiation zone and root hair; **(F–I)** 3-NO_2_-Tyr (100 μM, 2 h): **(F)** root apex; **(G)** meristematic zone; **(H)** transition zone; **(I)** differentiation zone, 24 h; **(J–N)** 3-NO_2_-Tyr (200 μM, 2 h): **(J)** general morphology of meristematic cells; **(K)** PI (propidium iodide) staining; **(L)** GFP-MAP4 signal; **(M,N)** MTs in epidermal cells of root apex. Images represent at least three independent experiments. Bar = 20 μm. Schematic drawings below the images represent MTs orientation relatively to the primary root or root hair axes: *interphase cortical MTs*: 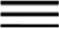 – transverse; 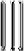, 
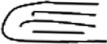
 – longitudinal; 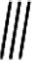 – oblique; 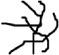 
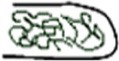
 – randomized; *interphase endoplasmic MTs:* 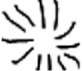 – radial; *mitotic MTs*: 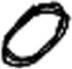 – preprophase band; 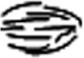 – mitotic spindle; 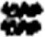 – phragmoplast (vertical arrow).

As the basic machinery required for plant growth and morphological responses is cytoskeleton, the changes in MTs organization after 3-NO_2_-Tyr treatment were studied. It was found that 3-NO_2_-Tyr at concentrations >50 μM causes the randomization of cortical microtubules (indicated by arrows) in epidermal cells of root apex (Figure 2F), transition and elongations zones (Figure 2H) as well as endoplasmic MTs in mer istematic cells (Figure 2G) in 2 h of treatment as compared to cells of control (HCl/L-Tyr-treated) seedlings (Figures 2A–I), where MTs kept their ordered orientation in each root growth zone (Figures 2A–E). MTs orientation and organization after 1, 5, and 10 μM 3-NO_2_-Tyr treatment remained unaltered.

In their same time, in (a)trichoblasts of the differentiation zone MTs retained oblique/longitudinal orientation (Figure 2I) as in cells of control (Figure 2E) that suggests a higher stability of MTs to 3-NO_2_-Tyr treatment as compared to MTs in transition and elongation zones (Figures 2C,D). Longitudinally oriented cortical MTs in elongating root hairs of control seedlings (Figure 2E) became randomized after >50 μM of 3-NO_2_-Tyr treatment for 24 h (Figure 2I, vertical arrow).

The increase 3-NO_2_-Tyr concentration to 200 μM (2 h) led to partial fragmentation and stabilization of MTs in epidermal cells of root apex (Figures 2M,N), while in treatment the cytoplasm shrinkage occurred in meristematic cells (Figures 2J,K) accompanied with the complete MTs depolymerization (Figures 2J,K, arrows).

### 3-nitrotyrosine localizes on microtubules of the intact by-2 cells

**Figure 3:**
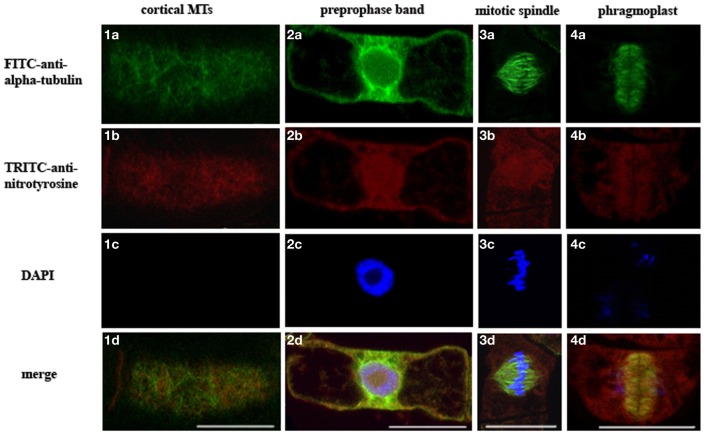
**Localization of 3-NO_2_-Tyr in interphase and mitotic microtubules of intact *N. tabacum* (BY-2) cells revealed by FITC-anti-α-tubulin (TU-01) and TRITC-anti-3-NO_2_-Tyr. (a)** TU-01 (green signal), **(b)** anti-3-NO_2_-Tyr (red signal) **(c)** DAPI staining (blue signal) **(d)** merge of images **(a–c)**. Types of MTs arrays: **(1)** cortical MTs; **(2)** preprophase band; **(3)** mitotic spindle; **(4)** phragmoplast. Bar = 20 μm.

To determine the presence of α-tubulin tyrosine nitration within interphase and mitotic MTs in BY-2 cells, we have performed double labeling of MTs using specific antibodies against α-tubulin (TU-01) and NO_2_-Tyr (anti-NO_2_-Tyr). Indirect immunofluorescent microscopy revealed that anti-3-NO_2_-Tyr antibodies decorated preprophase band [Figure 3(1b)], mitotic spindle [Figure 3(1c)] and phragmoplast [Figure 3(1d)], while no clearly colocalized signals from 3-NO_2_-Tyr and α-tubulin on cortical MTs was observed [Figure 3(1a)].

### NO Donor decreases the number of preprophase bands, mitotic figures and phragmoplasts in by-2 cells

**Figure 4:**
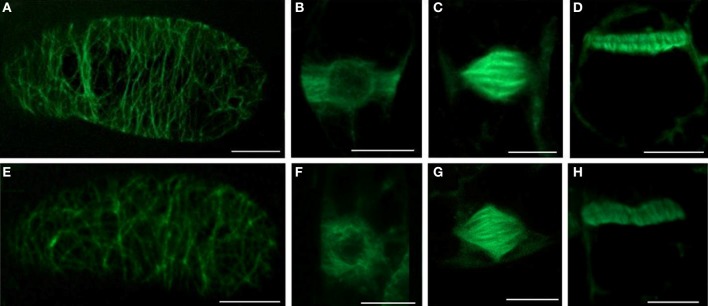
**The effects of SNP on the organization of different MTs arrays and on the number of mitotic MTs arrays in BY-2 (GFP-MBD) cells. (A–D)** control; **(E–H)** 5 mM SNP treatment during 3 h. Type of intrerphase and mitotic MT arrays: **(A,E)** cortical MTs; **(B,F)** preprophase band; **(C,G)** mitotic spindle; **(D,H)** phragmoplast. Bar = 10 μm.

The organization of cortical MTs (Figure 4E), preprophase bands (Figure 4F), mitotic spindles (Figure 4G), and phragmoplasts (Figure 4H) remained unaltered after 5 mM SNP treatment for 3 h and were comparable to control alignment (Figures 4A–D).

**Figure 5:**
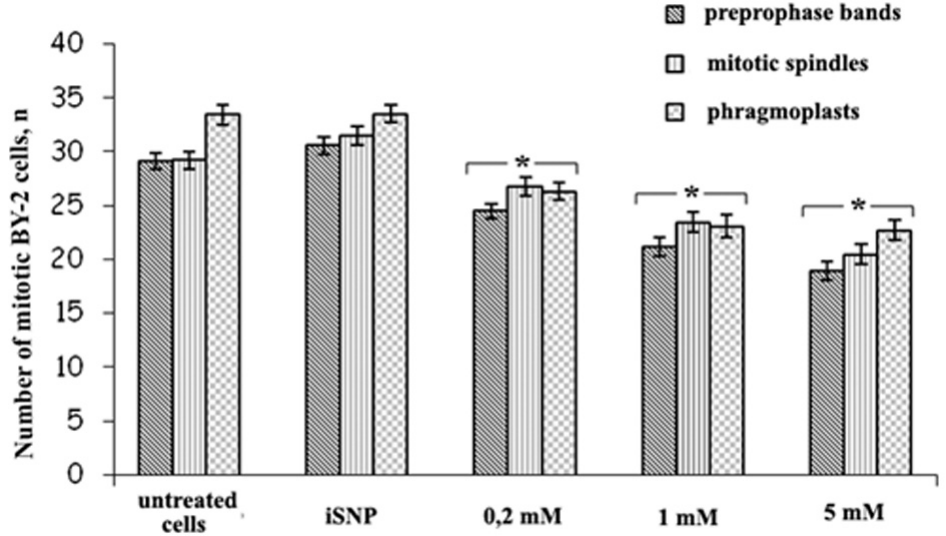
**The number of prepropase bands, mitotic spindles and phragmoplasts after SNP (200 μM, 1 and 5 mM) treatment for 3 h**. Light-inactivated SNP (iSNP) was used as a control. Cell number values are expressed as means ± *SE* (*n* = 1000 cells from four independent experiments). Vertical bars represent the *SE*. Columns with asterisk are significantly different (*t*-test, *P* < 0.001) from their controls (untreated and iSNP-treated cells). The number of GFP-labeled preprophase bands, mitotic spindles, and phragmoplats were double-checked by the number of pro-, ana-, meta-, and telophases revealed by DAPI.

However, SNP (200 μM, 1 and 5 mM) treatment decreased the number of mitotic arrays in BY-2 (GFP-MBD) cells (Figure 5). The most sensitive were the preprophase bands, while mitotic spindles and phragmoplasts number decreased almost to the same extent.

### *In silico* modeling of plant tubulin-kinesin-8 complexes

**Figure 6:**
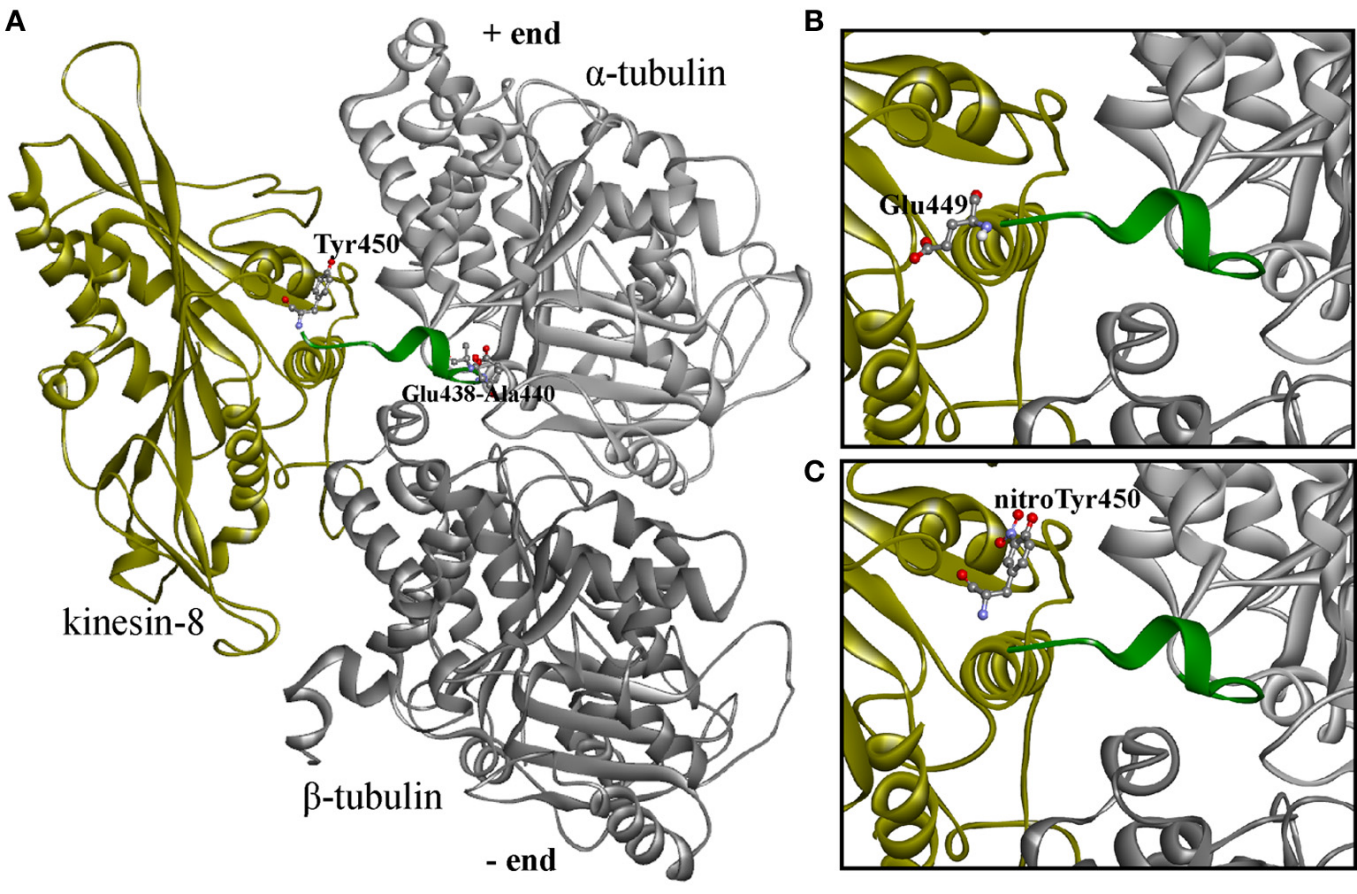
**3D-modeled plant tubulin-kinesin-8 complexes before the molecular dynamics simulations. (A)** Complex with non-modified α-tubulin; **(B)** complex with detyrosinated α-tubulin; **(C)** complex with nitrotyrosinated α-tubulin. Proteins shown as ribbons, amino acid residues—as balls and sticks colored by elements. Last 15 (14) residues colored by green are labeled terminated residues and residues with changed torsion angles.

In our previous work the reconstruction of tyrosinated, detyrosinated an tyrosine nitrated C-terminal region of α-tubulin from goosegrass *Eleusina indica* Gaertn. was reported (Blume et al., 2005), while in the present work we automatically modeled α- and β-tubulins from *Arabidopsis* with full C-terminal region used to design improved *in silico* system for nitrotyrosination studying. Primarily as a result of the imposition on the 1IA0 crystal (complex of KIF1A kinesin *Mus musculus* L. and β/α-tubulin interdimer *Sus scrofa* L.) constructed models of plant kinesin-8 and α- and β-tubulins with complete sequences were received a protein-protein complex. In this case, a short C-terminal α-helix of α-tubulin appeared to be sandwiched between tubulin and kinesin-8. However, since this spiral is not fixed in the 1IA0, it was assumed that it doesn't fall within the contact zone of kinesin with tubulin. Therefore, the C-terminal region in *Arabidopsis* α-tubulin from tubulin-kinesin-8 complex was removed from the space between the macromolecules by changing the torsion angles of amino acid residues in positions 438–440 (Figure 6A). The models of tubulins-kinesin-8 complex were also constructed with detyrosinated (Figure 6B) and nitrotyrosinated (on Tyr450) α-tubulins (Figure 6C).

The simulation of the molecular dynamics of these three complexes allowed us to analyze the behavior of α-tubulin C-terminal region depending on its modification by detyrosination or nitrotyrosination in contact with kinesin-8. Thus, *g_dist* module can calculate the distance between the centers of mass of two atomic groups as a function of time.

**Figure 7:**
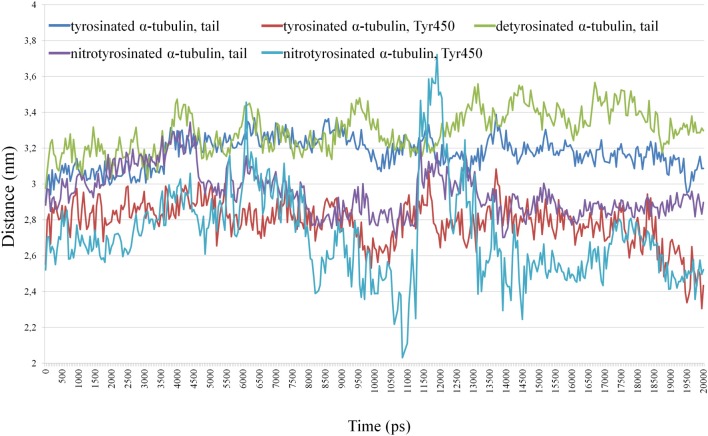
**Distance between the centers of masses of kinesin-8 and the C-terminal tails from last 15 (14) residues and non-modified and nitrated Tyr 450 α-tubulin**.

The distance is generated for the C-terminal tails from last 15 (or 14 in case of detyrosinated tubulin) amino acid residues and for non-modified and nitrated Tyr450 of α-tubulin in shown at Figure 7.

Tyrosine nitration (Tyr450) promotes greater convergence of C-terminus α-tubulin with kinesin as compared to detyrosinated and unmodified α-tubulins, and tail of detyrosinated α-tubulin has somewhat greater distance from the motor protein than tails from tyrosinated and nitrotyrosinated α-tubulins (see Supplementary Movies). Two peaks (3.4 and 3.7 nm) in the distance between the centers of mass of kinesin and terminal nitrated tyrosine (6.1 and 11.9 ns, respectively) indicate the presence of very short-term distance of modified residue from kinesin surface.

**Figure 8:**
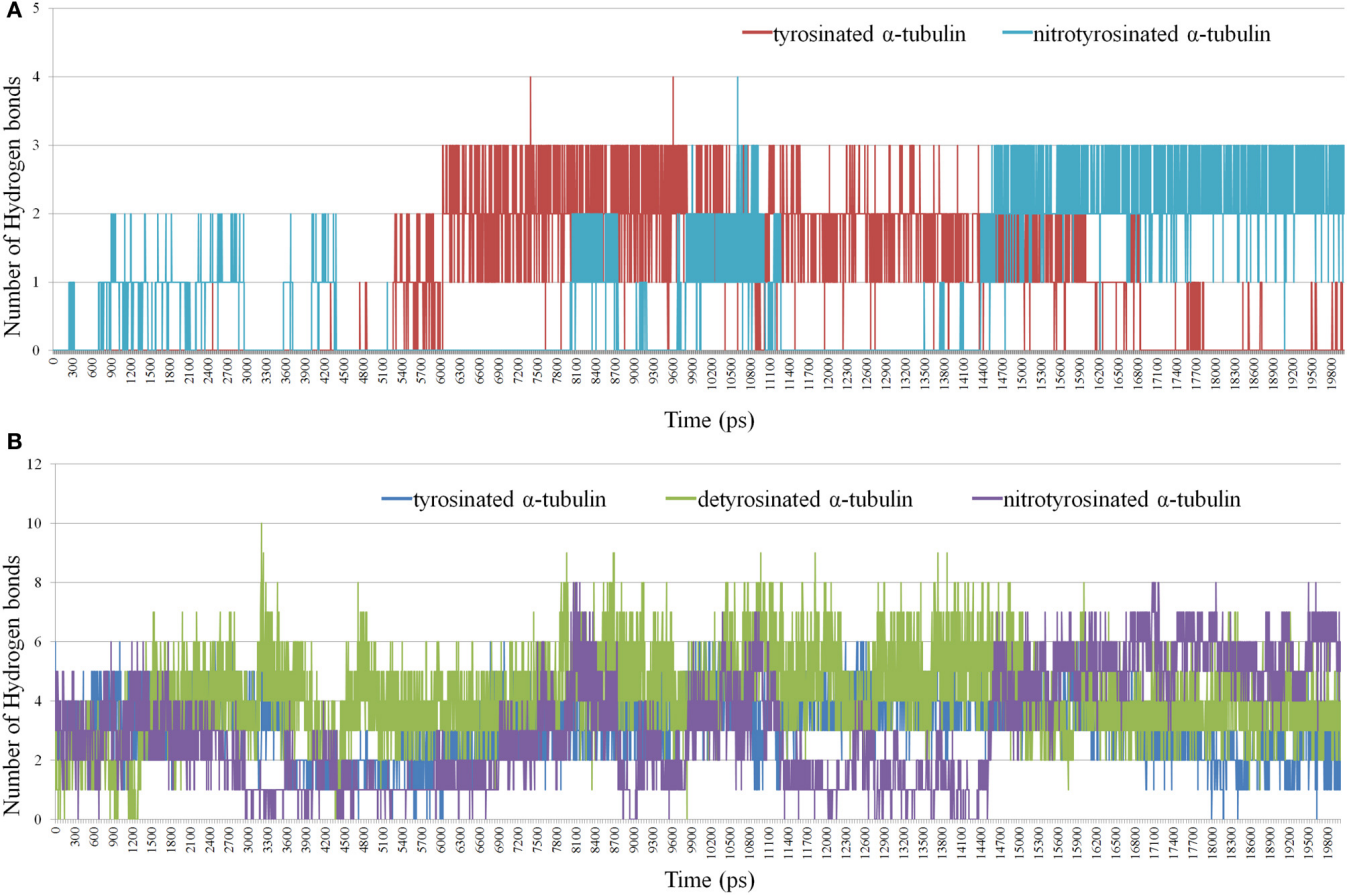
**Distribution of hydrogen bonds between kinesin-8 surface and different parts of α-tubulin. (A)** Hydrogen bonds in a case of terminated non-modified and nitrated Tyr 450; **(B)** Hydrogen bonds in a case of the last 15 (14) residues of α-tubulin.

In turn, the *g_hbond* module allows to compute and analyze the hydrogen bonds. Hydrogen bonds based on cutoffs for the angle Hydrogen–Acceptor (zero is extended) and the distance Donor–Hydrogen Acceptor–Acceptor were determined. OH and NH groups are regarded as donors, O is a temporary acceptor, N is an acceptor by default, but this can be switched. Dummy hydrogen atoms are assumed to be connected to the first preceding non-hydrogen atom. Logarithmic curves of hydrogen bonds distribution between the kinesin-8, unmodified and nitrated tyrosine 450 are shown in Figure 8A, indicate the best contact of the modified residue with the kinesin-8 surface, especially in the last 5 ns of dynamics.

Gaps in bond number at intervals of 5–8 and 11–14 ns, which coincide with two nitrotyrosine-specific residues peaks on *g_dist* graph, indicate the absence of any relation between the modified terminal amino acid and kinesin-8 molecule. It can be assumed that during the second gap, the terminal modified tyrosine gains optimal and advantageous position that is proved by the formation of two-three stable hydrogen bonds between it and the kinesin-8. Number of hydrogen bonds between the last 15 (14) residues of α-tubulin and motor protein (Figure 8B) also denotes the best contact between kinesin and tyrosine nitrated α-tubulin.

Thus, *in silico* modeling of plant kinesin-8 interaction with tubulin indicates the promoting of such posttranslational modification as terminal tyrosine residue nitrotyrosination within the α-tubulin a quite stable contact of tubulin C-terminal region with the kinesin-8 surface in region loop L12 and α4-helix.

## Discussion

As plant MTs and actin filaments reorganize in response to the increase of NO content *in vitro* and *in vivo* (Zhang et al., 2008; Kasprowicz et al., 2009; Shi et al., 2009; Yemets et al., 2009), it is accepted that they could play a role of NO sensors and targets in plant growth, development and plant-pathogen interactions. Herewith, α-tubulin and the dynamic F-actin act as downstream effectors of NO signaling cascades (Kasprowicz et al., 2009; Yemets et al., 2009, 2011). Fluctuations of NO levels provided by its exogenous donors (SNP, SNAP, GSNO, NO gas), scavenger (c-PTIO) and inhibitor of the mammalian NO-synthase (L-NAME, N^ω^-nitro-L-arginine methyl ester) affect *A. thaliana* primary root growth (Fernández-Marcos et al., 2011) and modulate MTs organization in root epidermal cells (Yemets et al., 2009, 2011). The existing data about the interrelation of RNS and MTs suggest that the organization of these cytoskeleton components could be regulated by both direct (tyrosine nitration/S-nitrosylation of tubulins and/or MAPs, microtubule-associated proteins) (Lindermayr et al., 2005; Landino et al., 2007; Yemets et al., 2011; Lozano-Juste et al., 2011) and indirect [e.g., modulation of cytosolic Ca^2+^ levels (Zhang et al., 2008)] mechanisms of NO signal transduction that are supposed to coexist in a plant cell dependent on local environment in the microcompartments (Zachgo et al., 2013), ROS/RNS balance (Livanos et al., 2012), cell/tissue/organ type (Yadav et al., 2013) and plant developmental stage (Begara-Morales et al., 2013).

Recent insights suggest that the original model of tyrosine nitration classically associated with RNS deleterious effects does not adequately capture the complexity of direct NO signaling in eukaryotic cells. In order to better understand the functional role of this modification, *A. thaliana* seedlings were exposed to different concentrations of 3-NO_2_-Tyr. It was found that 3-NO_2_-Tyr (5–200 μ M) inhibits *A. thaliana* roots growth in concentration-dependent manner (Figure 1A) as compared to L-Tyr/HCl-treated seedlings. Our results supports the findings of other authors claiming that the growing of rice seedlings in a medium supplemented with 20–500 μM 3-NO_2_-Tyr inhibited roots and coleoptiles growth in 70 and 60%, respectively (Jovanović et al., 2010). However, it was revealed that *A. thaliana* roots growth could be partially restored after the retransfer of 3-NO_2_-Tyr-treated seedlings (5 and 10 μM) to L-tyrosine containing medium (Figure 1A) supporting the assumption about the reversibility of tyrosine nitration and its dependency of 3-NO_2_-Tyr-concentration. Reversibility could be provided by the activity of the “nitrotyrosine denitrase” (Deeb et al., 2013), heme- and thiols-mediated non-enzymatic reduction of the nitrogroup to aminotyrosine with further removal by nitroreductases (Abello et al., 2009) or the precise cleavage of 3-NO_2_-Tyr from α-tubulin by TTCP (Bisig et al., 2002). Protein tyrosine nitration prevails throughout pea root development and progresses during its senescence as a wide distribution of 3-NO_2_-Tyr observed in epidermis, cortex, and vascular tissues of senescent roots (Begara-Morales et al., 2013).

Rice root growth inhibition was proposed to be explained by the delay or the complete block of mitotic cell division in meristematic zone (Jovanović et al., 2010). On the other hand, root growth is coordinated both by cell division and cell elongation, so it was advisably to study the effects of NO-2-Tyr on *A. thaliana* (GFP-MAP4) root cells elongation maintained by cortical MTs array. It was found that this modified aminoacid caused alteration of MTs organization in both epidermal and cortical cells of the transition and elongation (Figures 2B–D) as well as in meristematic zones (Figures 2B,C). The most susceptible to 3-NO_2_-Tyr were cortical MTs in epidermal cells of transition (distal elongation) zone, since the alteration of MTs organization (Figure 2H) of the transversely oriented array (Figure 2C) led to the swelling (loss of polar growth) of cells both of transition and elongation zones (Figure 1D). Generally, transition zone is the main signaling nexus in plant root and its cells are very active in the cytoskeletal rearrangements, endocytosis, endocytic vesicle recycling and electric activities (Baluška and Mancuso, 2013). It has to be also noted that the treatment of *A. thaliana* seedlings with 10–500 μM SNP revealed no impact on MTs organization in meristematic cells (Yemets et al., 2009).

It was established for the first time that 3-NO_2_-Tyr affects the process of root differentiation, since the treatment with 50-200 μM 3-NO_2_-Tyr for 24 h causes various morphological alterations of roots hairs (incurved, reverberant, crooked, bifurcated, etc.) with ceased growth (Figures 1C–M). Branching and/or bending of root hairs probably occur due to MTs depolymerization or stabilization in trichoblasts (Bibikova et al., 1999), while the cessation of elongation could be probably determined also by the randomization of the longitudinal MTs (Bibikova et al., 1999; Vassileva et al., 2005). Indeed, in >50 μM 3-NO_2_-Tyr-treated seedlings the randomization of MTs in trichoblasts of the differentiation zone was observed (Figure 2I). As cortical MTs guide tip growth of roots hairs and are in a generally longitudinal orientation along the shank of tip-growing cells (Van Bruaene et al., 2004; Vassileva et al., 2005; Rounds and Bezanilla, 2013), even the loss of their longitudinal orientation (randomization) is the sufficient requirement for the display of their altered morphology (Figures 1C–M). Indeed, root hair deformations in *Lotus japonicus* exposed to purified *M. loti* Nod factor could be due to the changes in microtubule configuration (Vassileva et al., 2005). Despite that for the initiation of tip growth of root hairs the reorganization of cortical MTs in trichoblasts from longitudinal/oblique to random is required (Van Bruaene et al., 2004), for further growth MTs have to regain longitudinal orientation (Vassileva et al., 2005). The cessation of further root hair growth might occur because MTs randomization persisted during the experiment. It has been also revealed that the alteration of tyrosine phosphorylation process (affected by the addition of protein kinases/phosphatases inhibitors) also leads to MTs randomization and disturbed root hairs morphology (Sheremet et al., 2012), what indicates the presumable interplay of tyrosine phosphorylation and tyrosine nitration of α-tubulin in the regulation of MTs properties. Taken together, these results suggest that 3-NO_2_-Tyr alters root hairs morphology and prevents their further growth, possibly due to MTs randomization. However, 3-NO_2_-Tyr effects on other mechanisms of apical growth, e.g., on the organization of actin microfilaments, have to be considered. Tyrosine nitrated proteins were abundant around the nuclei in the actively dividing cells of the adventitious root primordium (Yadav et al., 2013).

Another explanation of rice coleoptiles and root growth inhibition is cell cycle arrest in meristem cells (Jovanović et al., 2010). Cell division of cycling BY-2 cells was inhibited under low concentrations of 3-NO_2_-Tyr (50 nM–0.5 μM) possibly by irreversible α-tubulin tyrosine nitration leading to the impaired detyrosination and kinesins binding that disturbs phragmoplast function and cell plate formation (Jovanović et al., 2010). Though, 3-NO_2_-Tyr is localized on phragmoplasts [Figure 3(4d)] as well as on preprophase bands [Figure 3(2d)] and mitotic spindles [Figure 3(3d)] of the intact BY-2 cells under physiological conditions suggesting that this modification is not detrimental to the organization of these mitotic arrays. Furthermore, α-tubulin tyrosine nitration plays a key regulatory role in cell cycle progression of vascular smooth muscles cells because it has not induced necrotic or apoptotic death and caused cell cycle arrest at the G1/S boundary coincident with the decreased DNA synthesis (Phung et al., 2006). The breakdown of detyrosination/tyrosination cycle due to 3-NO_2_-Tyr incorporation into α-tubulin is expected to cause the predomination of short-time living MTs over long-time living ones as the dynamicity of α-tubulin C-terminus could be ranged from the maximum mobility from tyrosinated—tyrosine nitrated—detyrosinated (Blume et al., 2005).

It was also found that SNP treatment (200 μM, 1 and 5 mM) revealed no effects on MTs organization in BY-2 cells (Figure 5), however, induced dose-dependent decrease in the total number of the mitotic arrays. Such response might be a consequence of G_2_-M delay due to SNP influence on other mechanisms of cell cycle progression and check-points transition not related to cytoskeleton (Bai et al., 2012). SNP can stimulate the activation of cyclin expression, cell division and embryogenic cell formation in leaf protoplast-derived cells of alfalfa in the presence of auxin in a concentration-dependent manner (Ötvös et al., 2005).

In addition, cytoplasm shrinkage (Figures 2J,K) and MTs depolymerization (Figure 2L) were revealed after NO_2_-Tyr treatment at very high concentration (200 μM) as compared to control (200 μM L-Tyr) (Figure 2A), however, terminal deoxynucleotidyl transferase dUTP nick end labeling (TUNEL) assay is required to prove that the primary hallmarks of the PCD were actually observed. It is known that RNS production is required for PCD progression in plant cells (Clarke et al., 2000) and SNP (50 μ M) caused DNA damage revealed by TUNEL staining in *Arabidopsis* root cells (Bai et al., 2012). Though depolymerization of actin filaments because of the nitration of tyrosine residues induces apoptotic events in model lines of sickle cells (Aslan et al., 2003), the reciprocity of cytoskeleton reorganization and plant PCD progression has to be elucidated.

Our *in vitro* and *in vivo* findings are consistent with *in silico* modeling of the tyrosinated, detyrosinated and tyrosine nitrated plant α-tubulin complexes with the proteins from kinesin superfamily regulating MTs dynamical properties (Figures 6–8, Supplementary Movies 1–3). Members of kinesin superfamily of the motor MAPs use ATP hydrolysis energy for the spindle formation, cargo transport and regulation of spindle MTs dynamics. Both kinesin-8 and kinesin-13 families are involved into the control of mitotic spindle positioning and, hence, chromosome movement (Wiever and Walczak, 2011). Kinesin-8 family members are (+)-directed motor proteins (Peters et al., 2010). Moreover, kinesin KIF1A, the representative of kinesin-3 family, forms crystal complex with tubulins involved in anterograde transport (Kikkawa et al., 2001). Since MT (+)-end in growing axons is oriented toward the synapse, and (−)-end, respectively, toward the body of the neuron, KIF1A is (+)-directed motor protein. It has been suggested that the motor domains of both KIF1A and kinesin-8 contact with MT in the same way, and the L10-loop of kinesins oriented toward (+)-end. However, the majority of motor domains contacts with α-tubulin, and the surface of β-tubulin from the neighboring dimer in MT interacts with kinesin, where loops L2 and L11 are located (Kikkawa et al., 2001). In this paper we report for the first time that the interaction of kinesin-8 with detyrosinated α-tubulin C-tail is not as strong as with tyrosinated and tyrosine nitrated polymers (Figure 7) that might explain one of the putative mechanisms of the NO-mediated regulation of MTs dynamics. Furthermore, posttranslational tyrosine nitration of animal α-tubulin led to the decreased association of MT (+)-end with end-binding protein 1 (EB1) (Phung et al., 2006) that can also modulate eukaryotic MTs organization. Kinesin-1 preferentially binds to detyrosinated MTs rather than to tyrosinated ones, suggesting that the detyrosination/tyrosination of tubulin is likely part of kinesin-based microtubule depolymerizing activity (Peris et al., 2009). It was found recently that a kinesin-8 motor protein (Kip3) from budding yeast acts as a MT depolymerase conrolls microtubule sliding during mitotic spindle elongation in anaphase (Roostalu and Surrey, 2013). Sliding Kip3 activity promotes bipolar spindle assembly, while destabilizing one destabilizing inhibits spindle elongation and provides spindle disassembly that is required for normal spindle assembly (Su et al., 2013).

Tyrosine nitration of α-tubulins as the modification of detyrosination/tyrosination cycle extend the “tubulin code” aimed to distinguish different functional subpopulations of MTs and to adjust tubulin to MAPs and, consequently, the functions of MTs by inducing structural changes and modulating their activity, subcellular localization, stability, and interactions with other proteins and molecules (Parrotta et al., 2013). As single factor does not satisfactory explain the selectivity of α-tubulin tyrosine nitration, it can be supposed that other mechanisms with no relation to enzymatic incorporation of 3-NO_2_-Tyr into C-terminus also exist in plant cell. It should be noted that not only α-tubulin was defined as the target of tyrosine nitration, but also β-tubulin from rat liver cells (Aulak et al., 2001). In turn, *Arabidopsis* β-tubulin (β1, β2/3, and β4 chains) was found to be nitrated on tyrosine residues (Lozano-Juste et al., 2011). Besides tyrosine nitrated also S-nitrosylated α4 and α6 chains were detected by a biotin switch method in *Arabidopsis* (Lindermayr et al., 2005). Moreover, the effects of 3-NO_2_-Tyr on the actin filaments organization are also the point of interest.

In conclusion, on the one side, the increase of 3-NO_2_-Tyr content as a marker of tyrosine nitration is proved to be a reliable marker of the nitrosative stress, but from the other tyrosine nitration could be the regulatory modification of α-tubulin because: (1) basal level of *in vitro* α-tubulin tyrosine nitration exists in intact BY-2 cells (Yemets et al., 2011); (2) 3-NO_2_-Tyr decorates preprophase bands, mitotic spindles and phragmoplasts in the intact BY-2 cells (Figure 3); (3) *Arabidopsis* root growth inhibition induced by 5–10 μM 3-NO_2_-Tyr is partially reversible (Figure 1B); (4) 3-NO_2_-Tyr (5–100 μM) causes MTs randomization, not depolymerization, in *Arabidopsis* root cells; (5) α3/α5 and α6 chains of tubulin was found among the tyrosine nitrated proteins in *Arabidopsis* by a shotgun LC-MS/MS approach *in vivo* (Lozano-Juste et al., 2011); (6) 3D-reconstruction of *Arabidopsis* detyrosinated/tyrosinated/tyrosine nitrated α-tubulin - kinesin-8 complex as a model of the regulation of MTs dynamic properties; (7) denitrase and TTCP activities are expected to be found in plant cell.

Further work is needed to elucidate the possibility of tyrosine nitration to coexist with other RNS-mediated posttranslational modifications on the same tubulin molecule to generate MTs with combined functional properties. The studies of the reversibility of this posttranslational modification (via TTCP or denitrase) would also improve the existing knowledge about the direct NO signaling pathways. Recent progress in proteomic approaches is supposed to be aimed on the identification of the exact nitration sites and the compounding of protein profiles differentially modified by tyrosine nitration during plant development under eu-/distress conditions.

### Conflict of interest statement

The authors declare that the research was conducted in the absence of any commercial or financial relationships that could be construed as a potential conflict of interest.
